# 3cDe-Net: a cervical cancer cell detection network based on an improved backbone network and multiscale feature fusion

**DOI:** 10.1186/s12880-022-00852-z

**Published:** 2022-07-23

**Authors:** Wei Wang, Yun Tian, Yang Xu, Xiao-Xuan Zhang, Yan-Song Li, Shi-Feng Zhao, Yan-Hua Bai

**Affiliations:** 1 Key Laboratory of Carcinogenesis and Translational Research (Ministry of Education/Beijing), Department of Gynecologic Oncology, Peking University Cancer Hospital and Institute, Beijing, 100142 China; 2 School of Artificial Intelligence, Beijing Normal University, Beijing, 100875 China; 3 Key Laboratory of Carcinogenesis and Translational Research (Ministry of Education/Beijing), Department of Pathology, Peking University Cancer Hospital and Institute, Beijing, 100142 China

**Keywords:** Cervical cancer detection, Feature fusion, Backbone network, Adaptive anchors, Loss function

## Abstract

**Background:**

Cervical cancer cell detection is an essential means of cervical cancer screening. However, for thin-prep cytology test (TCT)-based images, the detection accuracies of traditional computer-aided detection algorithms are typically low due to the overlapping of cells with blurred cytoplasmic boundaries. Some typical deep learning-based detection methods, e.g., ResNets and Inception-V3, are not always efficient for cervical images due to the differences between cervical cancer cell images and natural images. As a result, these traditional networks are difficult to directly apply to the clinical practice of cervical cancer screening.

**Method:**

We propose a cervical cancer cell detection network (3cDe-Net) based on an improved backbone network and multiscale feature fusion; the proposed network consists of the backbone network and a detection head. In the backbone network, a dilated convolution and a group convolution are introduced to improve the resolution and expression ability of the model. In the detection head, multiscale features are obtained based on a feature pyramid fusion network to ensure the accurate capture of small cells; then, based on the Faster region-based convolutional neural network (R-CNN), adaptive cervical cancer cell anchors are generated via unsupervised clustering. Furthermore, a new balanced L1-based loss function is defined, which reduces the unbalanced sample contribution loss.

**Result:**

Baselines including ResNet-50, ResNet-101, Inception-v3, ResNet-152 and the feature concatenation network are used on two different datasets (the Data-T and Herlev datasets), and the final quantitative results show the effectiveness of the proposed dilated convolution ResNet (DC-ResNet) backbone network. Furthermore, experiments conducted on both datasets show that the proposed 3cDe-Net, based on the optimal anchors, the defined new loss function, and DC-ResNet, outperforms existing methods and achieves a mean average precision (mAP) of 50.4%. By performing a horizontal comparison of the cells on an image, the category and location information of cancer cells can be obtained concurrently.

**Conclusion:**

The proposed 3cDe-Net can detect cancer cells and their locations on multicell pictures. The model directly processes and analyses samples at the picture level rather than at the cellular level, which is more efficient. In clinical settings, the mechanical workloads of doctors can be reduced, and their focus can be placed on higher-level review work.

## Introduction

Cervical cancer is the fourth most common gynaecological malignancy globally. In 2018, approximately 570,000 new cases and 310,000 deaths occurred worldwide [1]. Traditional cervical cancer cell screening typically requires a pathologist to observe thousands of cells under a microscope and provide a report based on diagnostic criteria [2]. This approach is time-consuming, labour-intensive, heavily reliant on the doctor's experience and strongly subjective [3].

Computer-aided cervical cancer cell detection is likely to become a common clinical diagnosis approach for solving the above problems [4, 5]. According to whether the segmentation step is included in the analysis pipeline, the identification approaches for cervical cancer cells can be divided into segmentation-based recognition and object detection-based recognition methods. Segmentation-based recognition methods typically segment cells or cell components and then extract cell characteristics for cell classification [6–10]. The detection accuracy of such an approach typically depends on the results of cell segmentation, which makes it difficult to accurately identify overlapping cells with blurred cytoplasmic boundaries. Clinical applications face more difficulties. Object detection-based cervical cancer cell recognition has been a trend in recent years [11–13], and this technique uses an object detection framework based on a convolutional neural network (CNN) to obtain the classifications and locations of cancer cells. According to whether regional candidate boxes are generated, object detection methods can be divided into two categories: one-stage methods and two-stage methods [14].

Xiang et al. [12] proposed an automatic assisted cervical cell screening system based on You Only Look Once (YOLO)-v3-net and designed a classifier to further distinguish the categories of hard samples. Zhuang et al. [13] designed a special backbone network for cervical cancer cells and applied it to the single-shot multibox detector (SSD) framework. These deep learning-based cervical cancer cell detection algorithms are one-stage approaches [12, 13], and their detection accuracies are not high. Xu et al. [11] proposed a two-stage detection method and transplanted the Faster region-based CNN (R-CNN) [15] framework for natural images into cervical cancer cells. However, the difference between cervical cancer cells and natural images is not considered; as a result, the detection performance of this method is weak.

In this study, a two-stage cervical cancer detection algorithm based on an improved backbone network and multiscale feature fusion is proposed. In the first stage, cervical cell features with different sizes are extracted via an improved backbone network, and the feature extraction results can be verified through classification experiments. In the second stage, the location information of cervical cancer cells is obtained via a detection network. The features with different scales are fused through a feature pyramid, and then, adaptive anchors are located by K-means clustering. Additionally, a loss function that alleviates sample contribution imbalance is defined, which yields improved detection accuracy. The proposed approach directly processes and analyses samples at the picture level rather than at the cellular level, which is more efficient and meets clinical needs more effectively. Via a horizontal comparison of the cells on an image, the category information and location information of cancer cells can be obtained concurrently.

The primary contributions of this study are as follows:

- A two-stage cervical cancer detection network, namely, 3cDe-Net, is proposed, and it can detect small cancer cells with different sizes and ratios.
- A cervical cell feature extraction network is designed, and dilated convolution and group convolution are introduced in the backbone and incorporated into a deep residual network. The proposed network avoids upsampling operations and reduces the loss of small-cell information on the feature map.
- The anchor frame size and ratio are adaptively determined by K-means clustering, which is more suitable for cervical cancer cells and can provide better prior knowledge. A loss function is redefined to address the imbalance between negative and positive samples for cervical cancer cell detection.

## Related works

### Backbone network

Currently, deep learning-based detection algorithms must typically identify the features of an input picture through a feature extraction network. The feature extraction network used for the classification task is also known as the backbone network. The Visual Geometry Group Network (VGGNet) [16] is the backbone network of Faster R-CNN [15], and its structure is simple. ResNet uses a deeper network structure to extract more complex features [17]. Both networks are still relatively common backbone networks. In addition, DenseNet densely connects each network layer with other layers [18] and DetNet is specifically designed for object detection [19].

Existing backbone networks are primarily used to recognize natural images. However, in cervical cell images, the canceration of cells is a gradual change process, making it difficult to distinguish normal cells from cancerous cells using traditional backbone networks. Additionally, the scales of cervical cells vary, and small cells are difficult to identify on deeper feature maps, which makes it more difficult to detect these small cells. Thus, group convolution is used to enhance the expression ability of the extracted features. Concurrently, to better distinguish normal cells from abnormal cells, a dilated convolution is used to improve the resolution of the generated map and the classification accuracy achieved for small cells. Furthermore, this convolution operation also reduces the number of calculations caused by upsampling during feature map fusion.

### Object detection based on deep learning

Compared with classification, detection involves an additional location task. Therefore, based on the backbone network, a network called a detection head should be added to locate the object region proposal. Thus, the backbone network and the detection head together construct the detection network. According to whether the detection head contains a region proposal network (RPN), the available detection networks can be divided into one- and two-stage methods. One-stage detection methods do not generate region proposals, and the location and category prediction processes are completed in one stage. However, the accuracies of these methods could be improved. Two-stage methods first perform predetection by generating regional proposals and then fine-tuning the location and classification processes, yielding high accuracies. Faster R-CNN [15] is a classic two-stage detection network and is effective for many natural image datasets [20], but the network is not suitable for cervical cells due to the fact that anchors are generated based on natural images. In addition, the small cells retain less information on the feature map, which affects the detection accuracy achieved for these cells. Finally, the loss function of the network does not consider sample size imbalance. To solve these problems, feature pyramids are used to integrate deep and shallow feature maps, as they integrate deep semantic features and shallow location information more effectively than other approaches [21]. Therefore, small cells can be detected, and adaptive anchor boxes can be generated. Furthermore, a new loss function can be defined, improving the detection accuracy of cervical cancer cells.

## Proposed methodology

**Fig. 1:**
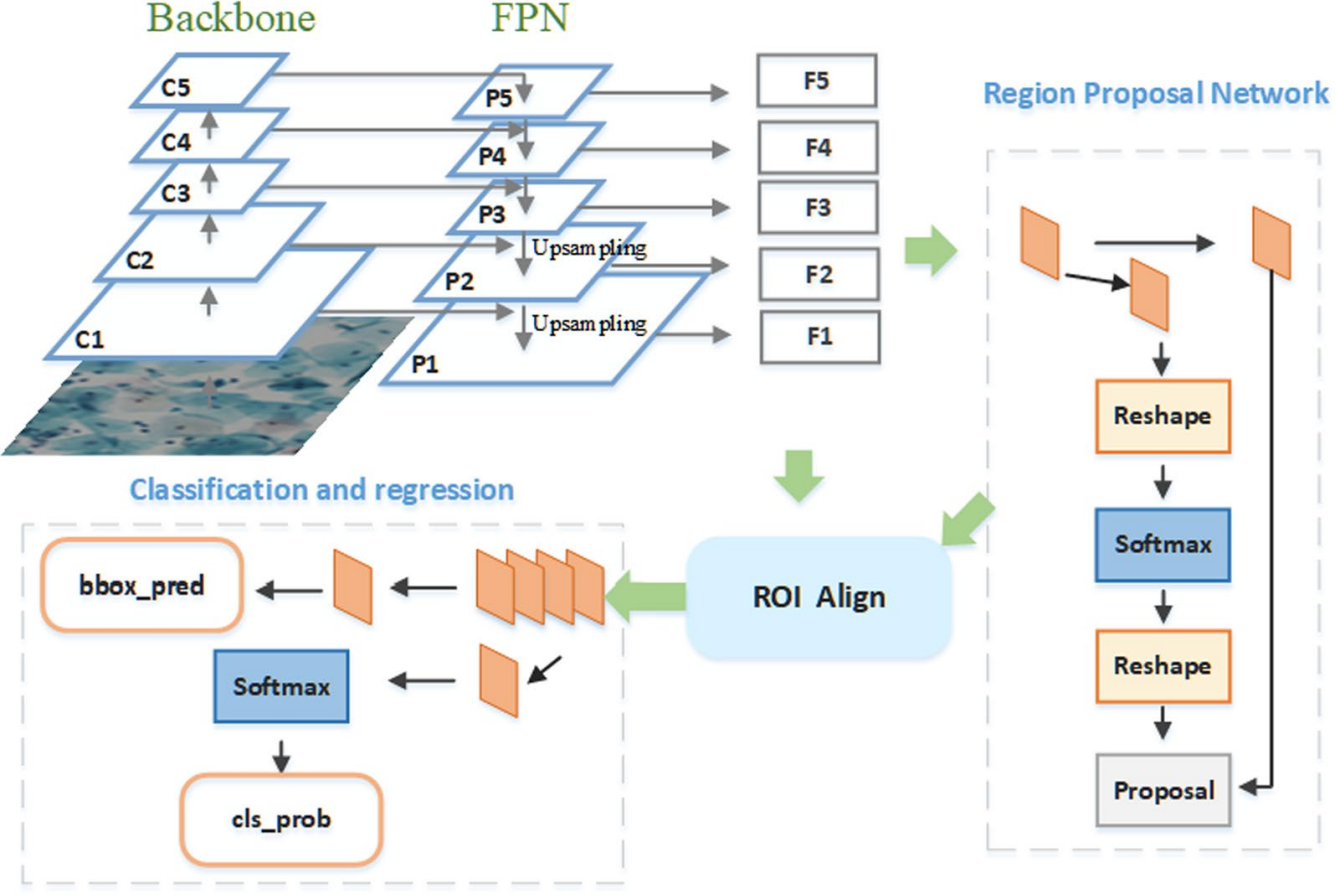
Overall flow of the proposed 3cDe-Net

The overall framework of the proposed network is shown in Fig. 1. The backbone network uses a dilated convolution ResNet (DC-ResNet). The algorithm first performs cancer cell predetection through an RPN and then obtains the results through a classification and regression network.

Multiple feature maps with different scales are generated from the backbone network. Feature fusion is performed via a feature pyramid network (FPN) to obtain the final predicted feature map, and then, this feature map is fed to the RPN. The FPN achieves feature fusion by upsampling to form deep and shallow feature maps with the same dimensions, and it can transfer deep semantic features to shallow layers to supplement the available semantic information. As a result, high-resolution and strong semantic features, which are capable of detecting small objects, are obtained. The RPN first adaptively generates anchor boxes on the generated prediction feature maps and then selects and adjusts these anchor boxes to obtain better region proposals. Next, the proposals and feature maps are fed into the classification and regression network. Finally, cervical cancer cells are predicted and located.

### Improved backbone network: DC-ResNet

**Fig. 2:**
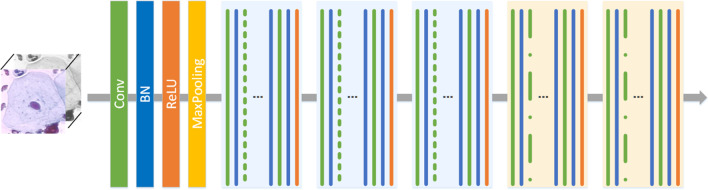
Network structure of the proposed DC-ResNet

DC-ResNet, derived from ResNet, is an improved backbone network. In DC-ResNet, a dilated convolution and a group convolution are introduced, and the details of the network structure are shown in Fig. 2. The input images are successively fed to the network through its convolutional layer (Conv), batch normalization layer (BN), rectified linear unit (ReLU) activation function and pooling layer (MaxPooling), and then, feature maps are obtained via multiple convolution groups [22]. The first three groups (blue) use residual grouping convolution, and the last two groups (yellow) use residual dilated convolution.

To improve the feature expression ability of the network, each feature map is fed into the fully connected layer to obtain the score of the predicted category. Each fully connected layer is followed by a dropout layer to prevent overfitting.

**Fig. 3:**
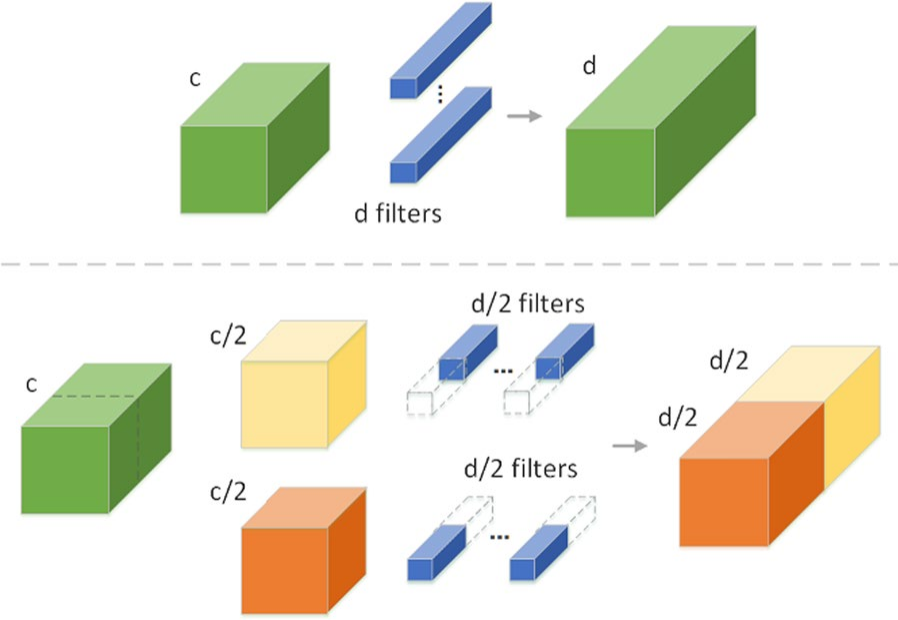
Group convolution

Group convolution was originally a training method [23] that was designed to solve hardware resource limitations. To obtain more distinguishable cervical cancer cell features, the convolutional divisions on the channels are grouped, and then, the results of each group are *concatenated*. The hyperparameter problem is solved by grouping convolution. Thus, the model accuracy is improved without increasing the number of parameters. The convolution operation is performed by multiple GPUs, and the calculation results are connected. The calculation process is shown in Fig. 3, where *c* is the dimensionality before convolution, and *d* is the dimensionality after convolution.

**Fig. 4:**
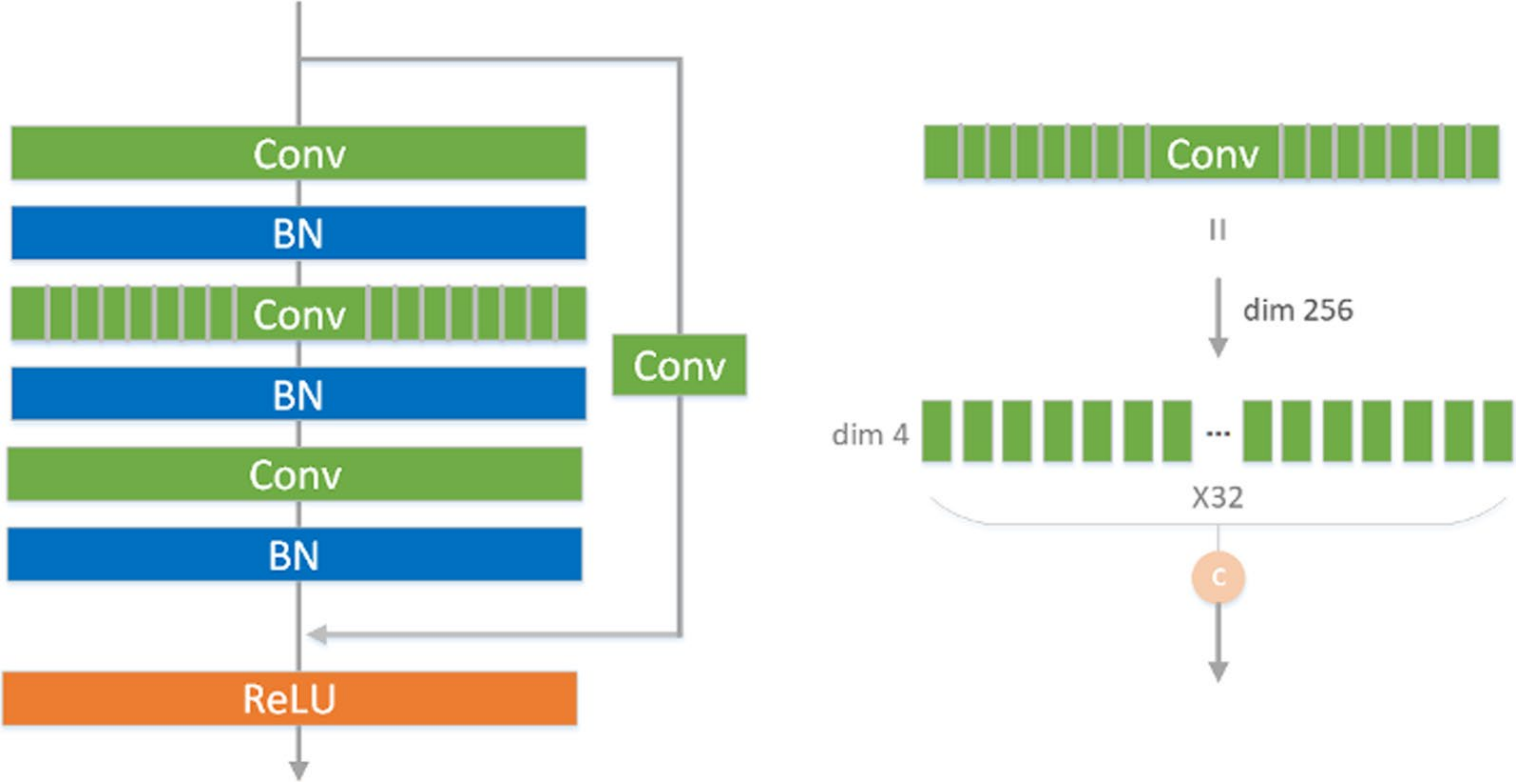
Residual group convolution block

In this study, residual group convolution, which is based on residual networks, is introduced, and the details are shown in Fig. 4. The left panel is the overall structure diagram, and the right panel is the detailed diagram of the group convolution operation.

Residual hole convolution performs hole convolution on the residual network. The calculation formula for obtaining the output feature size of the hole convolution is as follows:

1 $$\begin{array}{*{20}c} {y = \frac{{x - k - \left( {k - 1} \right) \times \left( {d - 1} \right) + 2p}}{s} + 1} \\ \end{array}$$

where *y* is the size of the output feature map, *x* is the size of the input image, *k* is the size of the convolution kernel, *d* represents dilation, *p* is the padding, and *s* is the stride. The feature maps before and after convolution have the same parameters.

**Fig. 5:**
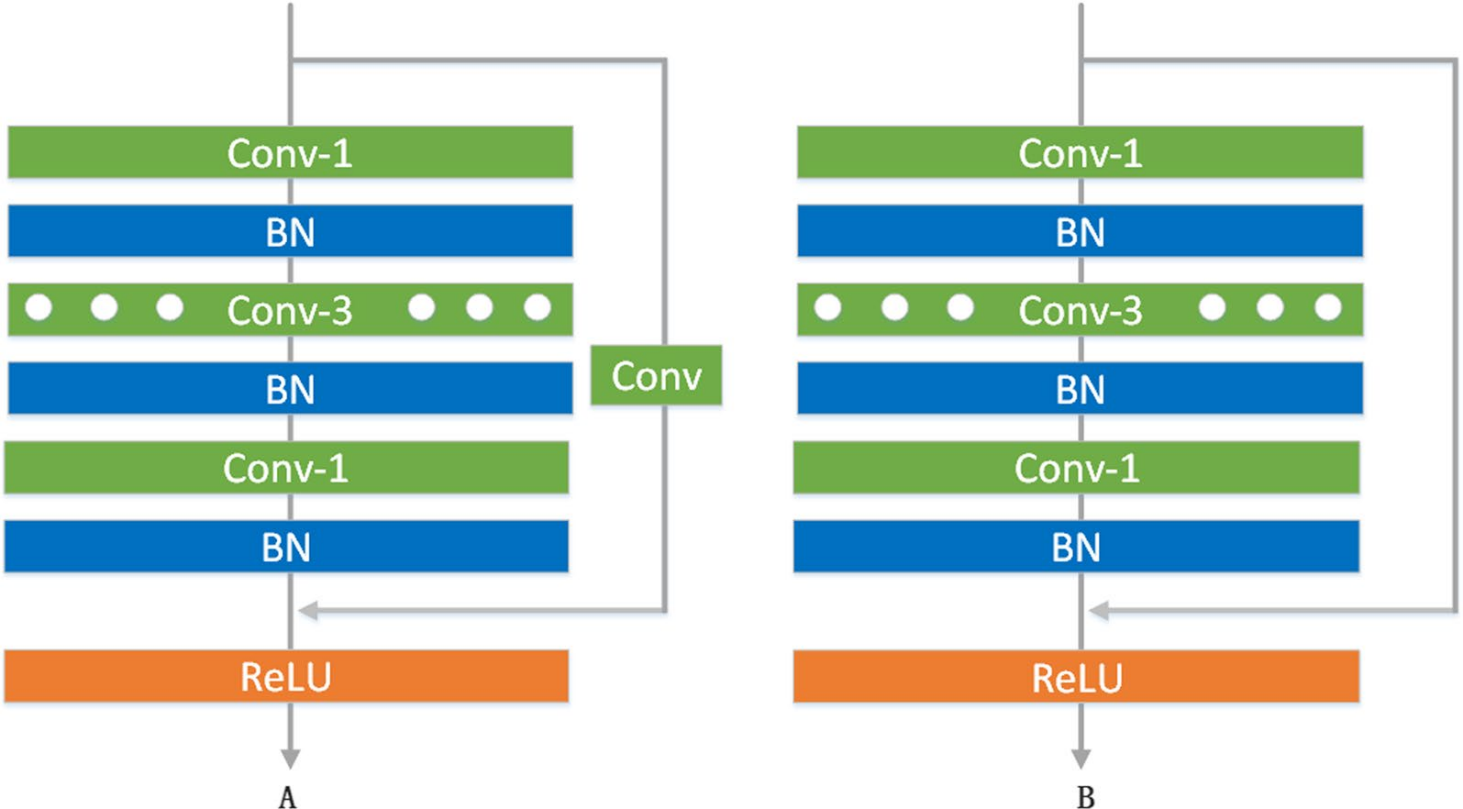
Residual dilated convolution block

The residual dilated convolution structure is shown in Fig. 5. The overall structure is similar to that of residual group convolution, but dilated convolution is used instead of group convolution. The size of the feature map obtained after the dilated convolution is unchanged. The residual dilated convolution operation has two structures A and B. The difference is whether the residual branch contains an added 1 × 1 convolution.

Downsampling/upsampling operations are not required for feature fusion. Thus, the size of the image feature map is larger than that of the original image. Therefore, the small cells at each feature point are more informative, and more edge information is retained.

### Generation of anchor boxes

**Fig. 6:**
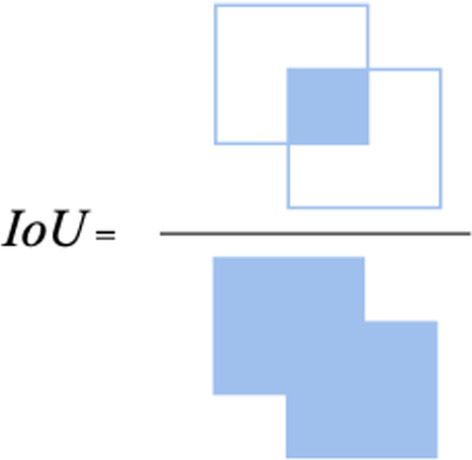
IoU calculation diagram

When the RPN generates region proposals, anchor boxes with different sizes at different feature layers of the FPN can be generated. The sizes and ratios of the anchor boxes are typically set based on prior domain knowledge or datasets. However, in this study, the changes in the sizes and ratios of cancer cells are large, which creates a challenge for anchor box generation. Inspired by YOLO v2 [24], the best *k* boxes can be obtained by K-means clustering. The locations of all cells are not certain. The sizes and ratios of anchors are measured by looking at all the target boxes as if they are located at the origin. When clustering is performed, the distances between the prediction boxes and centres are calculated by Eq. (2):

2 $$\begin{array}{*{20}c} {b\left( {box,center} \right) = 1 - IoU\left( {box,center} \right)} \\ \end{array}$$

where the intersection over union (IoU) represents the fitting degree of the two boxes and is the ratio of the area of the intersection and the union between the predicted box and the real box, as shown in Fig. 6.

The deep feature maps are small, and the receptive field is large, which is good for large cells. The shallow feature maps are the opposite, making them more suitable for detecting small cells. Therefore, anchors with different sizes and proportions can be generated on each feature map.

### Definition of the loss function

The detection network is a multitask learning model that must predict the classifications and locations of cervical cancer cells. Therefore, the loss function should include classification and regression losses, and its definition is as follows:

3 $$\begin{array}{*{20}c} {L\left( {\left\{ {P_{i} } \right\},\left\{ {bbox_{i} } \right\}} \right) = \frac{1}{{N_{cls} }}\mathop \sum \limits_{i} L_{cls} \left( {p_{i} ,p_{i}^{*} } \right) + \lambda \frac{1}{{N_{reg} }}\mathop \sum \limits_{i} P_{i}^{*} L_{reg} \left( {bbox_{i} ,bbox_{i}^{*} } \right)} \\ \end{array}$$

where $\mathop \sum \limits_{i} L_{cls} \left( {p_{i} ,p_{i}^{*} } \right)$ is the classification loss, $p_{i}$ is the real category, and $p_{i}^{*}$ is the predicted category. The classification function is calculated using cross-entropy loss, and $\lambda$ is the weight that balances the two task losses. $\mathop \sum \limits_{i} P_{i}^{*} L_{reg} \left( {bbox_{i} ,bbox_{i}^{*} } \right)$ represents the regression loss and only calculates positive samples; it does not include negative samples. The $smooth_{L1}$ function is used to calculate the regression loss. The definitions of these losses are as follows:

4 $$\begin{array}{*{20}c} {L_{reg} \left( {bbox_{i} ,bbox_{i}^{*} } \right) = \mathop \sum \limits_{i \in x,y,w,h} smooth_{L1} \left( {bbox_{i} - bbox_{i}^{*} } \right)} \\ \end{array}$$

5 $$\begin{array}{*{20}c} {smooth_{L1} \left( x \right) = \left\{ {\begin{array}{*{20}c} {0.5x^{2} } & {if\;\left| x \right| < 1} \\ {\left| x \right| - 0.5} & {otherwise} \\ \end{array} } \right.} \\ \end{array}$$

Setting the weights $\lambda$ to balance the classification effect and the positioning loss remains challenging. The parameter $\lambda$ is usually set manually. However, when performing the position regression task, the model considers the samples with regression losses greater than 1 more when λ increases because the loss of the regression task is unconstrained. Therefore, when designing the loss function, more consideration should be given to those samples with losses that are less than 1. Inspired by Pang et al. [25], the original $smooth_{L1}$ is replaced with $balanced_{L1}$, which is defined as follows:

6 $$\begin{array}{*{20}c} {balanced_{L1} \left( x \right) = \left\{ {\begin{array}{*{20}c} {\frac{\alpha }{b}\left( {b\left| x \right| + 1} \right)\ln \left( {b\left| x \right| + 1} \right)} & {if\;\left| x \right| < 1} \\ {\gamma \left| x \right| + C} & {otherwise} \\ \end{array} } \right.} \\ \end{array}$$

where α is used to control the gradient changes exhibited by samples with losses of less than 1 and γ is used to adjust the upper limit of the error. By adjusting the above two parameters, it is possible to balance the gradient contribution of each sample.

## Experiments and results

### Datasets and evaluation metrics

**Fig. 7:**
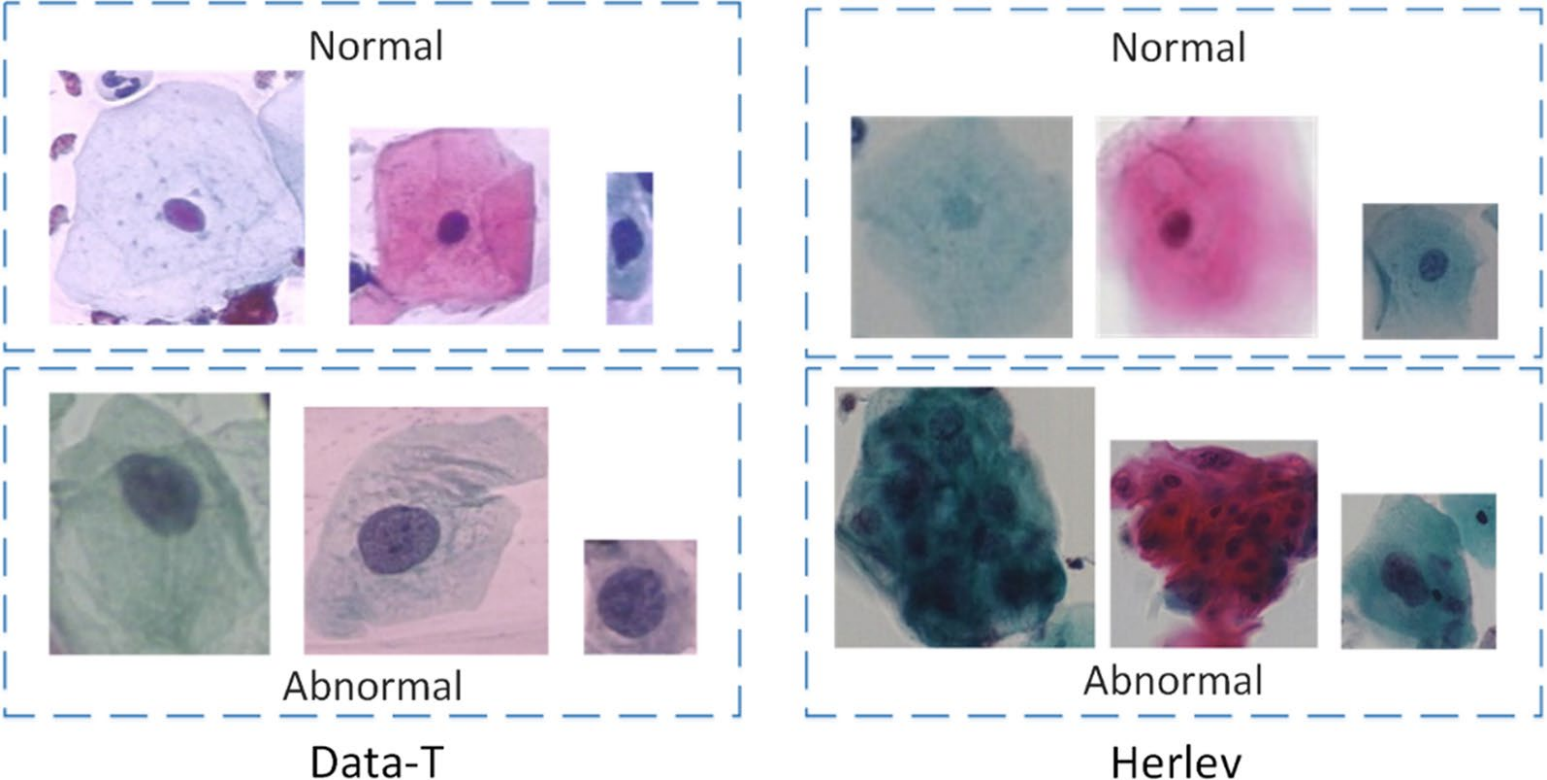
Example images from the two datasets

The experimental data used for evaluation in this study are derived from the Tian-chi competition dataset (Data-T)^1^ and the Herlev dataset.^2^ Figure 7 shows several images from these datasets. In Data-T, each cervical cell smear image contains multiple cervical cells, which can be used for classification and detection. In the Herlev image dataset, each image contains only one cervical cell, which can be used only for classification.

#### Data-T

This dataset was obtained from the preliminary data of the Cervical Cancer Risk Diagnosis Intelligent Challenge and contained 800 thin-prep cytologic test (TCT) images labelled by a professional pathologist, including 500 positive images and 300 negative images. Positive pictures were used to label the locations of abnormal squamous epithelial cells. Due to the large sizes of the original pathological images (each image was approximately 40,835 × 42,371 pixels), each original pathological image was divided into several images with 800 × 800 pixels to facilitate processing. Thus, 6627 abnormal squamous epithelial cells were obtained. A negative sample refers to an image that does not contain cervical cancer cells.

The dataset samples only contained the labelled locations of abnormal squamous epithelial cells. In this study, 6627 normal squamous epithelial cells were screened from 300 negative pictures using semisupervised learning methods, and together with 6627 positive samples, a total of 13,254 sample images were described by a cervical cancer cell classification dataset. The images were divided into training, validation and test sets according to a ratio of 8:1:1, and the ratio of positive to negative samples was 1:1. To train a model with good generalizability, the sample images were augmented by operations including rotation and flip transformation.

#### Herlev

This dataset contains images of cervical cancer cells that were collected by Herlev University Hospital in Denmark. It includes 917 single-cell images with 200 × 100 pixels, including 242 normal cells and 675 abnormal cells. This dataset has become the primary study dataset for the classification of cervical cancer cells.

Due to the small amount of available data and the imbalance between the positive and negative samples, the sample images were first augmented by centre rotation and translation operations. The normal cell images were rotated 20 times, and the abnormal cell images were rotated 10 times. As a result, the numbers of positive and negative samples were approximately equal. Finally, the training set, verification set, and test set were divided at a ratio of 8:1:1.

In the classification experiments, metrics including sensitivity, specificity, h-mean, F1 measure and accuracy were used to evaluate the performance of the proposed feature extraction network. Sensitivity represents the proportion of correct images among all predicted cancer cell images, and specificity represents the proportion of correct images among all predicted normal cell images. The results of the detection experiment were evaluated by the mean average precision (mAP) metric.

### Network parameters and implementation details

The sizes of the input images of the backbone network were 224 × 224 × 3, and the details of the network structure and parameters of DC-ResNet are listed in Table 1.

**Table 1:** Structure and parameters of DC-ResNet

Improved backbone: DC-ResNet			
$7 \times 7$, 64, stride 2			
$3 \times 3$, max pooling, stride 2			
Residual group convolution	$$\left[ {\begin{array}{*{20}c} {1 \times 1} & {128} \\ {3 \times 3} & {128} \\ {1 \times 1} & {256} \\ \end{array} } \right] \times 3$$		Group 32
	$$\left[ {\begin{array}{*{20}c} {1 \times 1} & {256} \\ {3 \times 3} & {256} \\ {1 \times 1} & {512} \\ \end{array} } \right] \times 4$$		Group 32
	$$\left[ {\begin{array}{*{20}c} {1 \times 1} & {512} \\ {3 \times 3} & {512} \\ {1 \times 1} & {1024} \\ \end{array} } \right] \times 6$$		Group 32
Residual dilated convolution	$$B:\;\left[ {\begin{array}{*{20}c} {1 \times 1} & {1024} \\ {3 \times 3} & {256} \\ {1 \times 1} & {1024} \\ \end{array} } \right] \times 1$$	$$A:\;\left[ {\begin{array}{*{20}c} {1 \times 1} & {1024} \\ {3 \times 3} & {256} \\ {1 \times 1} & {1024} \\ \end{array} } \right] \times 2$$	Dilation 2 Stride 2
	$$B:\;\left[ {\begin{array}{*{20}c} {1 \times 1} & {1024} \\ {3 \times 3} & {256} \\ {1 \times 1} & {1024} \\ \end{array} } \right] \times 1$$	$$A:\left[ {\begin{array}{*{20}c} {1 \times 1} & {1024} \\ {3 \times 3} & {256} \\ {1 \times 1} & {1024} \\ \end{array} } \right] \times 2$$	Dilation 2 Stride 2
fc-1024			
fc-256			
fc-2			

Experiments were performed on a workstation with the Ubuntu 16.04 operating system and a 12-GB NVIDIA GeForce 2080Ti GPU. While training the backbone network, the stochastic gradient descent (SGD) optimization algorithm was used to optimize the model parameters. The batch size was set to 32. The learning rate of each layer was initially set to 0.01. After 50 epochs of training, the learning rate was reduced to 1/10 every 10 epochs. The momentum was set to 0.9, and training ended after 1000 epochs. While training the detection network, the SGD optimization algorithm was again used to optimize the model parameters. The batch size was set to 6, the learning rate of each layer was initially set to 0.00125, and the learning rate was reduced to 1/10 after 16 epochs and 22 epochs. The momentum was set to 0.9, and the weight was decayed by 0.001.

### Results and analysis

On the Data-H dataset, ResNet-50 and ResNet-101 were used as baselines for comparison with the proposed DC-ResNet. Table 2 lists the quantitative comparison results and shows that the proposed backbone network (DC-ResNet) performed better than the baselines in terms of all metrics except for sensitivity. However, specificity is more important than sensitivity for the detection of cervical cancer cells due to the fact that the majority of cervical cell samples are normal.

**Table 2:** Quantitative comparison obtained on the Data-H dataset

Method	H-means (%)	Sensitivity (%)	Specificity (%)	F1 (%)	Accuracy (%)
ResNet-50	96.82	96.68	96.98	96.82	96.83
ResNet-101	96.75	97.12	96.37	96.76	96.75
DC-ResNet	97.11	95.92	98.34	97.09	97.13

The proposed DC-ResNet has 59 convolutional layers, and ResNet-50 has 50 convolutional layers. To verify the validity of the structure of the proposed DC-ResNet, we compared DC-ResNet with ResNet-101 (with 101 convolutional layers). Table 2 shows that DC-ResNet outperformed the other models. Additionally, all evaluation metrics achieved by the ResNet-101 network, except for sensitivity, were lower than those of ResNet-50, which may have been caused by the use of a limited number of datasets. Although no fittings of these complex models occurred, they did not necessarily produce better results.

**Fig. 8:**
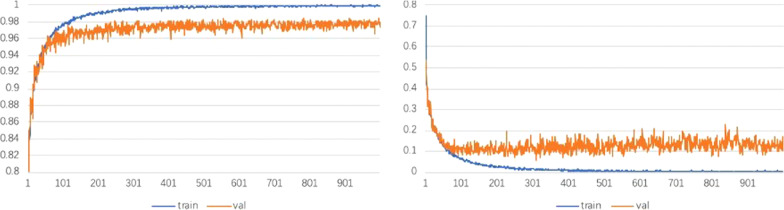
Accuracy and loss curves of DC-ResNet

**Fig. 9:**
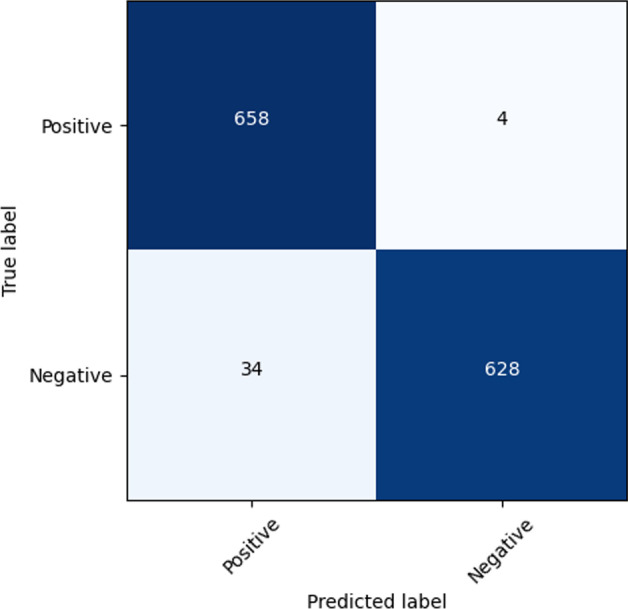
Confusion matrix of DC-ResNet

Figure 8 shows the accuracy and loss curves produced by the DC-ResNet backbone network on the training and validation sets. A total of 1000 epochs were used for training, and after each epoch, the training effect was verified on the validation set. The model was basically fitted for approximately 500 epochs. The final validation set loss fluctuated around 0.1, and the accuracy fluctuated around 98%. Figure 9 shows the confusion matrix yielded by DC-ResNet on the test set. From the confusion matrix, we can see that our model can achieve high performance on classification tasks, especially for negative samples.

**Fig. 10:**
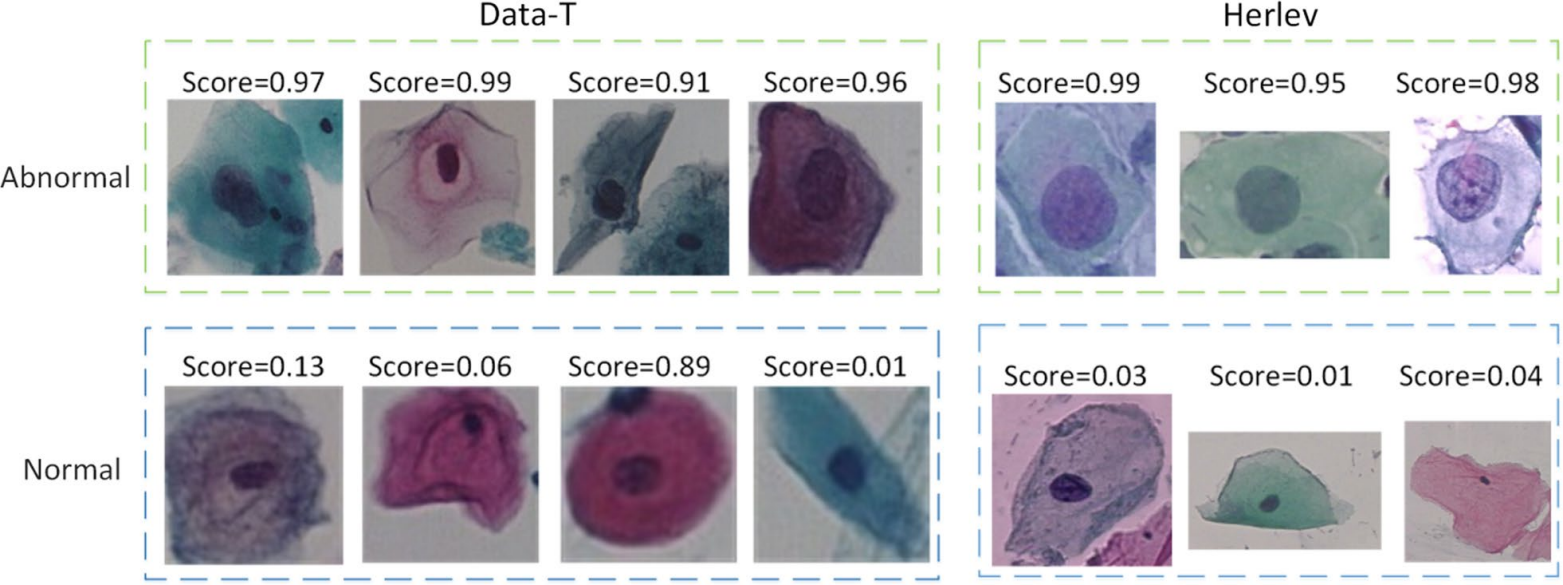
Examples of correct recognition results obtained by DC-ResNet

On the Herlev dataset, we used Inception-v3 [26], ResNet-152 [17] and a feature concatenation network [27] as baselines for comparison with the proposed DC-ResNet. The details of the quantitative comparison are listed in Table 3, which shows that the proposed DC-ResNet achieved the highest classification accuracy by nearly 4%. Due to the small amount of data in the Herlev dataset, the fivefold cross-validation method was used to verify the proposed network. The results indicated that the proposed DC-ResNet was superior to the baselines in terms of accuracy and exhibited better stability. The partial recognition results obtained by DC-ResNet on the two datasets are shown in Fig. 10.

**Table 3:** Quantitative comparison on the Data-H dataset

Method	Accuracy
Inception-v3 [25]	89.66 ± 1.89%
ResNet-152 [25]	90.87 ± 1.48%
Feature concatenation [22]	92.63 ± 1.68%
DC-ResNet	96.7% ± 1.1%

Table 4 lists the mAPs of the detection networks with different improvement measures, and the performance of the proposed 3cDe-Net was best, with a maximum mAP of 50.4%. The reasons for the performance improvement achieved by the proposed model are the anchor obtained by K-means clustering, the improved loss function, and the replacement of the backbone network with DC-ResNet. The mAP metric increased by 3% due to improvements in the detection network and the backbone network. The average IoU increased by 5% due to the anchor size generated by K-means clustering, and the mAP increased by 0.8% based on the incorporation of the generated anchor ratio into the detection network.

**Table 4:** Detection results of 3cDe-Net

Improved anchor	Improved loss	DC-ResNet	mAP@0.5 (%)
			47.3
√			48.1
√	√		49.3
√	√	√	50.4

**Fig. 11:**
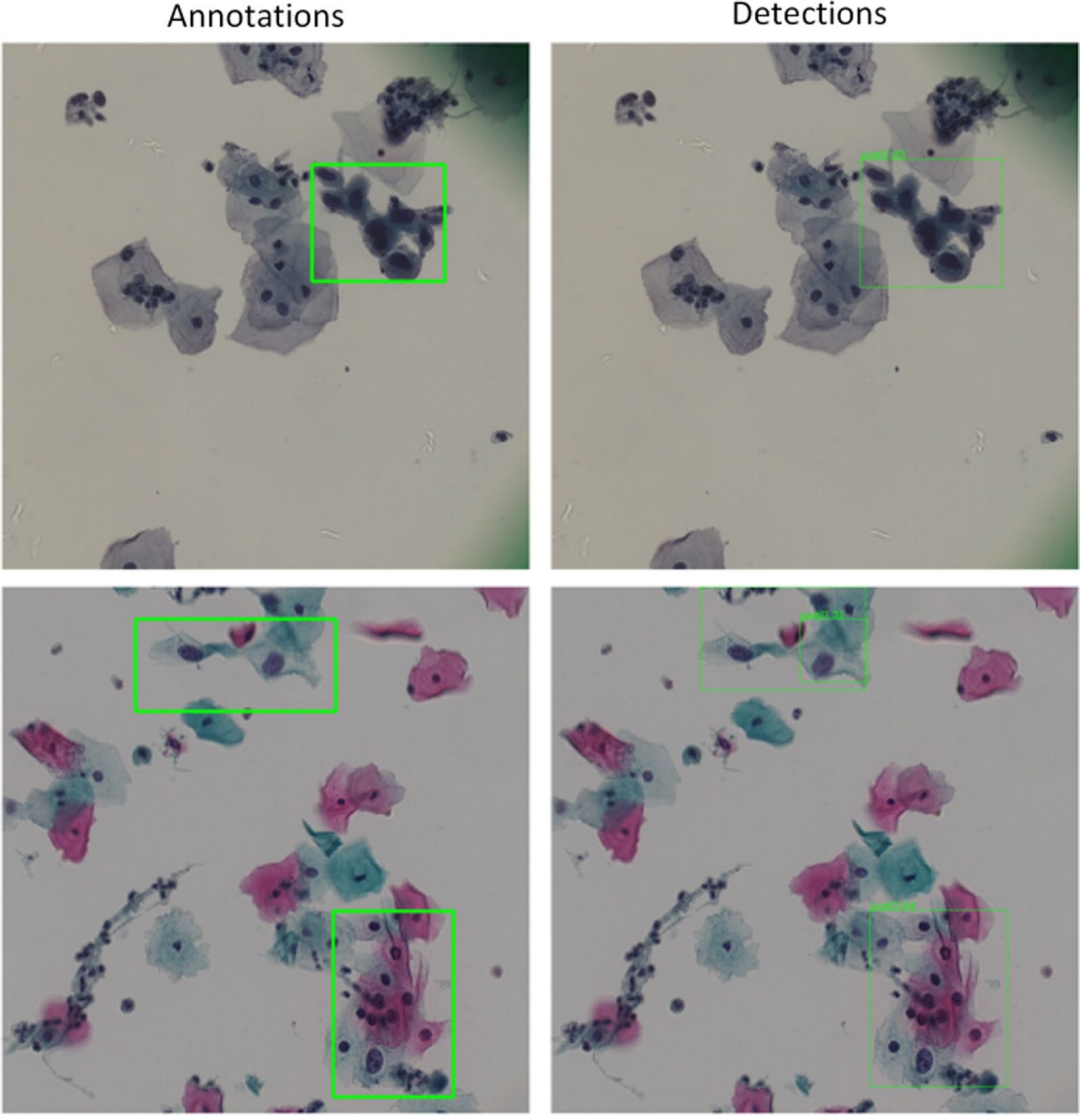
Detection examples of 3cDe-Net

When utilizing the improved $balanced_{L1}$, the mAP increased by 1.2% with $\alpha = 0.5$ and $\gamma = 1.5$. By replacing the backbone network (the original ResNet-50) with DC-ResNet, the mAP increased by 1.1%. Several detection results obtained by our 3cDe-Net are shown in Fig. 11.

## Discussion

In this work, we proposed 3cDe-Net based on a feature extraction network (DC-ResNet) for detecting cervical cells. Since the currently employed feature extraction network was designed for natural image datasets (such as ImageNet), it cannot be effectively adapted to cervical cell images. In cervical cell images, the cells are closely distributed, varying in size and ratio according to two morphologies (single cells and cell clusters). In view of the above characteristics, on the basis of a deep residual network, residual hole convolution was used to obtain features with larger receptive fields of view and higher resolutions, and group convolution was used to obtain a model with better expression ability.

To verify the effectiveness of DC-ResNet with respect to the detection of cervical cancer cells, we performed a comparative experiment with ResNet-50 and ResNet-101 in terms of the mAP indicator, and the results are listed in Table 5. The mAP of ResNet-101 was not higher than that of ResNet-50, which shows that simply increasing the complexity of the model may lead to model overfitting on small datasets. Concurrently, the mAPs of DC-ResNet-50 were at least 1% higher than those of ResNet-50, showing that the improved performance of the proposed model was not achieved due to the increase in the number of network layers but rather the effects of the network structure changes.

**Table 5:** mAP results of different backbone networks

Backbone	mAP@0.5 (%)	mAP@0.75 (%)
ResNet-50	45.4	26.2
ResNet-101	45.5	25.9
DC-ResNet	46.7	26.5

We also analysed the influence of the number of feature maps of DC-ResNet. F[1,2,3,4], F[1,2,3,5], F[1,2,4,5] and F[1,2,3,4,5] represent the feature fusion layers of the FPN in the corresponding feature layers in Fig. 1. The detection effects achieved by feature fusion with different combinations are shown in Table 6. The worst detection effect was 46% when the input feature map was numbered [1–4]. Although the number of feature maps was reduced, the mAP was still 5% higher than that of ResNet. This result indicates that the superior performance of the proposed model is due to the increase in the number of feature layers and the structure of the proposed DC-ResNet itself.

**Table 6:** Results of FPN fusion with feature maps from different layers

The number of fusion feature map	mAP@0.5 (%)
1, 2, 3, 4	46.0
1, 2, 3, 5	46.5
1, 2, 4, 5	46.1
1, 2, 3, 4, 5	46.7

Furthermore, in the proposed detection network, the detection head based on Faster R-CNN was improved, and different anchor boxes could be automatically set for different target sizes. Additionally, targets with different sizes and ratios were able to be predicted on feature layers at different depths.

In brief, 3cDe-Net is a two-stage detection method that avoids upsampling operations and reduces the loss small cell information on the feature map. The YOLO-v3-based method proposed by Xiang et al. [12] and the SSD-based method proposed by Zhuang et al. [13] are one-stage detection methods that have higher detection efficiency. However, these one-stage methods do not generate regional candidate boxes, and the prediction of locations and classes is completed in one stage, so the accuracy degrades.

Cervical cancer cell images are different from natural images. Clinical pathological images contain thousands of cells with highly complex cell conditions. A detection method for cervical cancer cells must consider various conditions, such as whether single cells are present, whether the cells overlap, etc., and the accuracy is greatly affected. The Faster R-CNN-based method proposed by Xu et al. [11] does not fully take these factors into account, so its detection effect is not good. In this work, some improvements were made to the model based on the characteristics of cervical cancer cell images. The semantic information of the observed deep features was added to the shallow features through a feature pyramid to achieve multiscale feature fusion; the K-means clustering method was used to obtain anchor frame sizes and ratios that were more suitable for cervical cancer cells and to provide better prior knowledge. To minimize the regression loss, a new balanced L1-based loss function was developed to reduce unbalanced sample contribution losses. The accuracy and efficiency of the proposed detection method were verified by cervical cancer cell detection experiments. The experimental results demonstrated that the performance of the proposed 3cDe-Net was best. However, our method can be further improved regarding the detection of negative samples, and the false detection rate for negative samples needs to be reduced in the future.

## Conclusion

In this paper, a cervical cancer cell detection network, namely, 3cDe-Net, was presented. The network is based on an improved backbone network and multiscale feature fusion, and it consists of the backbone network and a detection head. On the one hand, a feature extraction network DC-ResNet was designed for cervical cells. On the basis of the deep residual network, residual hole convolution was used to obtain features with larger receptive fields of view and higher resolutions, and group convolution was used to attain better feature expressiveness. Then, multiscale feature fusion was realized through an FPN. On the other hand, the detection head based on Faster R-CNN was improved, and the sizes and ratios of the cervical cell anchor frames were adaptively determined by K-means clustering. Furthermore, a loss function was redefined to address the imbalance between negative and positive samples for cervical cancer cell detection. Experiments on the Data-T and Herlev datasets illustrated that the proposed model outperformed existing methods and achieved a mAP of 50.4%. The next step in this field of research is to identify the types and stages of cancer cells based on the identification of cervical cancer cells.

## Data Availability

The datasets analysed during the current study are available in the Tian-chi Initiative and Herlev. https://tianchi.aliyun.com/competition/entrance/231757/introduction. http://mde-lab.aegean.gr/index.php/downloads

## Footnotes

1 1 https://tianchi.aliyun.com/competition/entrance/231757/introduction

2 2 http://mde-lab.aegean.gr/index.php/downloads
